# Management of Hydrocoelom in an Axolotl (*Ambystoma mexicanum*)

**DOI:** 10.1002/vms3.70373

**Published:** 2025-05-19

**Authors:** Şeyma Sueda Cirit, Şinasi Borgahan Deniz, Burhan Cirit, Abdulahad Bayraktar

**Affiliations:** ^1^ Cerrahi Anabilim Dalı Ankara Üniversitesi Veteriner Fakültesi Ankara Türkiye; ^2^ Ankara Üniversitesi Sağlık Bilimleri Enstitüsü Ankara Türkiye; ^3^ Centre for Host Microbiome Interactions King's College London London UK; ^4^ Biyoistatistik ve Tıp Bilişimi Anabilim Dalı İstanbul Üniversitesi‐Cerrahpaşa Cerrahpaşa Tıp Fakültesi Istanbul Türkiye

**Keywords:** axolotl, *Ambystoma mexicanum*, amphibian, coelomic cavity, hydrocoelom

## Abstract

A 3‐year‐old axolotl (*Ambystoma mexicanum*) presented with anorexia, weakness and abdominal swelling due to hydrocoelom. According to the anamnesis, the symptoms had occurred 3 months prior. The radiographic and ultrasonographic examinations revealed that these symptoms resulted from the fluid accumulating in the coelomic cavity. Then, the habitat of the axolotl and the fluid in the coelomic cavity were inspected. Then, the patient's hydrocoelomic fluid was drained and medical treatment was performed. In addition, habitat arrangement and diet were changed. At the follow‐up visit 1 week later, the patient reported a significant decrease in hydrocoelom and a significant increase in appetite and mobility. Laboratory results showed no microbiologic growth in the sample taken from the coelomic cavity. The patient remained healthy during a 1‐year follow‐up. The last follow‐up examination revealed a significant decrease in all of the symptoms. In conclusion, non‐infective hydrocoelom was observed in an axolotl, and medical treatment was performed.

## Graphical abstract

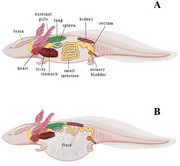

## 1 Introduction

*Ambystoma mexicanum*, also known as axolotl, is a cold‐water species native to the Lake Xochimilco region in the Valley of Mexico. Water conditions in Lake Xochimilco between 1978 and 1988 were estimated to be between 16°C and 20°C and pH between 7.4 and 8.0 (Farkas and Monaghan 2015; Ortiz‐Ordoñez et al. 2016).

**FIGURE 1:**
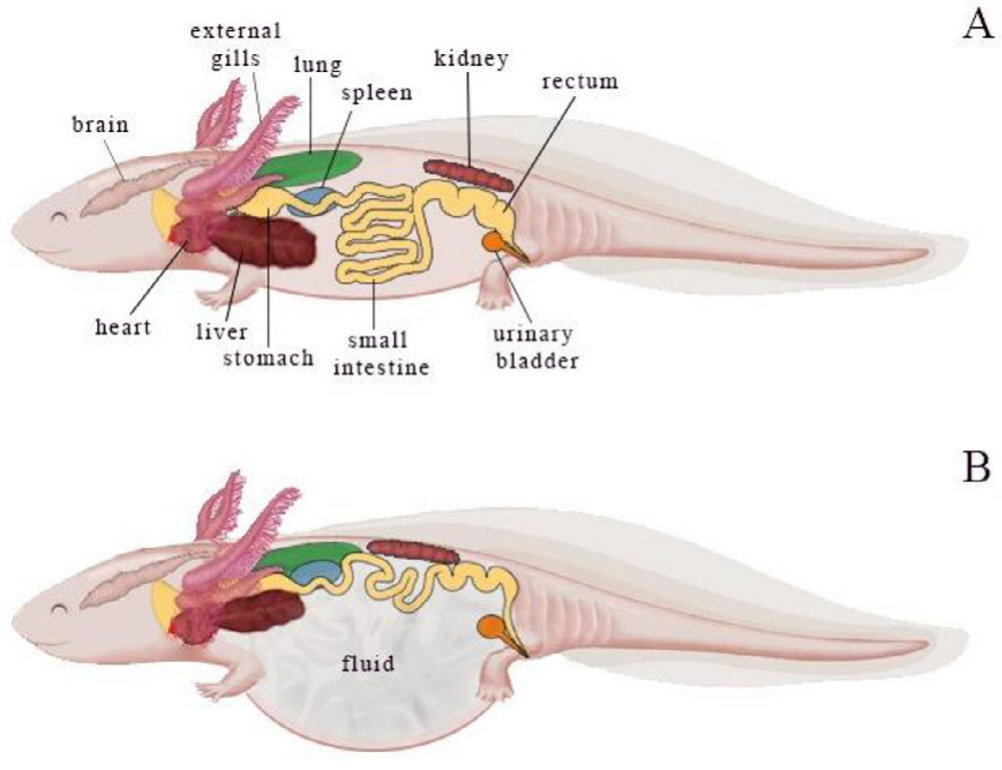
Anatomical view of the internal organs of (A) a healthy axolotl and (B) a sick axolotl with hydrocoelom formation.

Axolotls have permeable skin with protective mucosa (Canadian Council on Animal Care 2011a; Farkas and Monaghan 2015). Although the thorax and abdomen are not separated by a diaphragm, coelomic cavities exist which are located with internal organs (Figure 1). Axolotls with a length of over 20 cm from the tip of the snout to the cloaca and older than 10 months are classified as adults (Canadian Council on Animal Care 2011a; Vieu et al. 2024). Male axolotls are more elongated than females and exhibit a cloacal projection (Reiß et al. 2015).

Axolotls have a highly primitive immune system (Farkas and Monaghan 2015; Gentz 2007). An inadequate immune system causes the organism to be more affected by the stress factors to which it will be exposed. Microorganisms, changes in the external environment (e.g., increase in water temperature), pollutants (e.g., chlorine, ammonia), pH disruption and trauma (e.g., attack by other axolotls and manual intervention) are considered as the main stress factors (Baitchman 2008; Farkas and Monaghan 2015; McMenamin and Hadly 2010; Ortiz‐Ordoñez et al. 2016). Habitat and feeding arrangements were made for a week until the analysis results were available. Axolotl, like most aquatic amphibians, has a permeable skin that allows water loss or absorption (Baitchman 2008). This skin permeability causes the axolotl to be directly affected by changes in water. Toxins released by the decomposition of faeces, waste feed, other organic matter and changing water pH can cause diseases. Good pH is important for mucosa formation, which preserves skin (Baitchman 2008). Therefore, it was recommended to change the existing aquarium water by 20% daily and to collect feed residues a few hours after feeding. In addition, pH should be kept at 7.0–7.5, water should be free from chlorine and chloramines, and temperature should be kept at 14°C–20°C (Smith 2011). Treatment protocols for patient amphibians will be inadequate unless appropriate environmental conditions are provided for these ectothermic creatures (Sykes and Greenacre 2006).

Contrary to many other amphibians, axolotls are not capable of metamorphosis unless administered with thyroid hormones (Page and Voss 2009). These animals, which spend most of their lives in water, meet 40%–60% of their oxygen needs by surface respiration due to their developed lungs. In addition, they can also respire through their gills and skin via cutaneous gas exchange (Farkas and Monaghan 2015). Poor water quality due to inadequate water changes or poor filtration is the biggest stress factor for axolotls in captivity (Bjorklund and Duhon 1996). These conditions can lead to decreased reproduction, reduction in size, dry skin, dehydration and even death (McMenamin and Hadly 2010).

Hydrocoelom is the accumulation of fluid in the coelomic cavity due to various reasons (Baitchman 2008; Borland 2000; Clancy et al. 2015; Del‐Pozo et al. 2011). According to a pilot study, all clinically healthy axolotls have a small quantity of fluid in the coelomic cavity, but it is physiologically distinct from hydrocoelom (Vieu et al. 2024). Studies have shown that infectious causes, renal diseases and circulatory disorders are frequently involved in the aetiology of the disease (Canadian Council on Animal Care 2011a; Clancy et al. 2015; Del‐Pozo et al. 2011; Nace et al. 1974). However, the priority should be investigating environmental factors. Poor water quality and inappropriate rearing conditions (e.g., toxic ammonia, nitrite or nitrate levels) are chronic stressors for axolotls and are of vital importance (Bjorklund and Duhon 1996). Axolotls with hydrocoelom also display abdominal swelling, weakness and anorexia. Regular general health checks, follow‐ups and early initiation of treatment for domestic axolotls are important for case success (Del Valle and Eisthen 2019; James MacHale and Hedley 2021).

The purpose of this case report is to describe the diagnosis and treatment processes of a hydrocoelom case in an axolotl who was brought to our clinic with abdominal swelling. Moreover, this study aims to be a reference for veterinarians in terms of amphibian diseases by contributing to the existing literature.

## 2 Case Story

**FIGURE 2:**
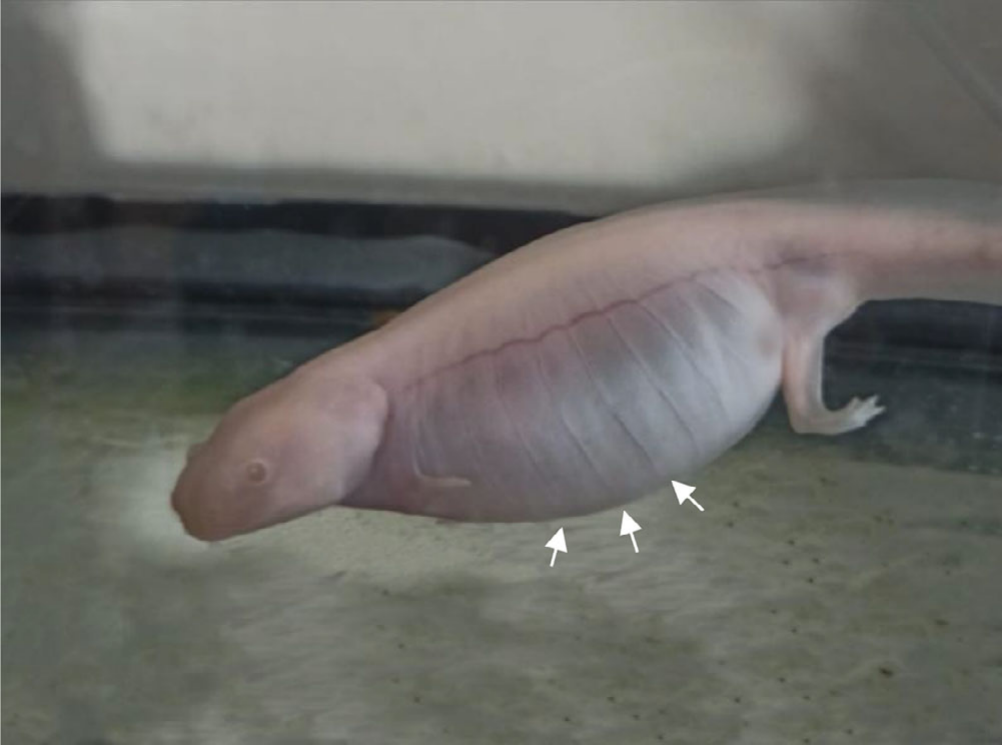
Image of the patient axolotl when brought to the clinic (white arrows).

A 3‐year‐old axolotl was presented with abdominal swelling and associated malaise for 3 months (Figure 2). It had no visible injuries. On clinical examination, it was observed that the aquarium water was purified tap water, and the temperature and pH were not controlled by the owner. Furthermore, the right anterior and posterior legs of the patient had been digested by another axolotl. The owner reported that the aquarium volume and water level of the axolotl were insufficient, and water cleaning and lighting were irregular. The patient was fed with axolotl feed and bloodworms available in the market.

**FIGURE 3:**
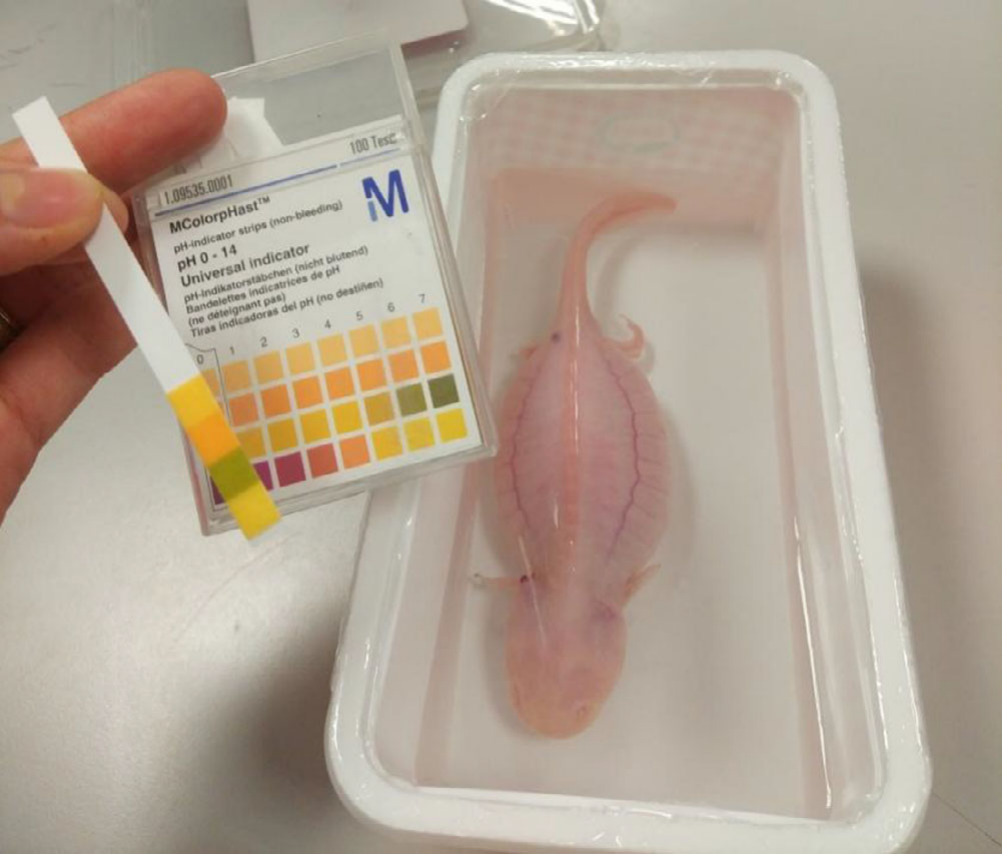
Measurement of pH 6 of the fluid sample taken from the living area at the first examination.

In the samples taken from the patient's living area, the water temperature was 24°C and the pH was 6 when measured with Mcolorphast pH Indicator Strips (Figure 3). The pH of tap water was measured as 5.

**FIGURE 4:**
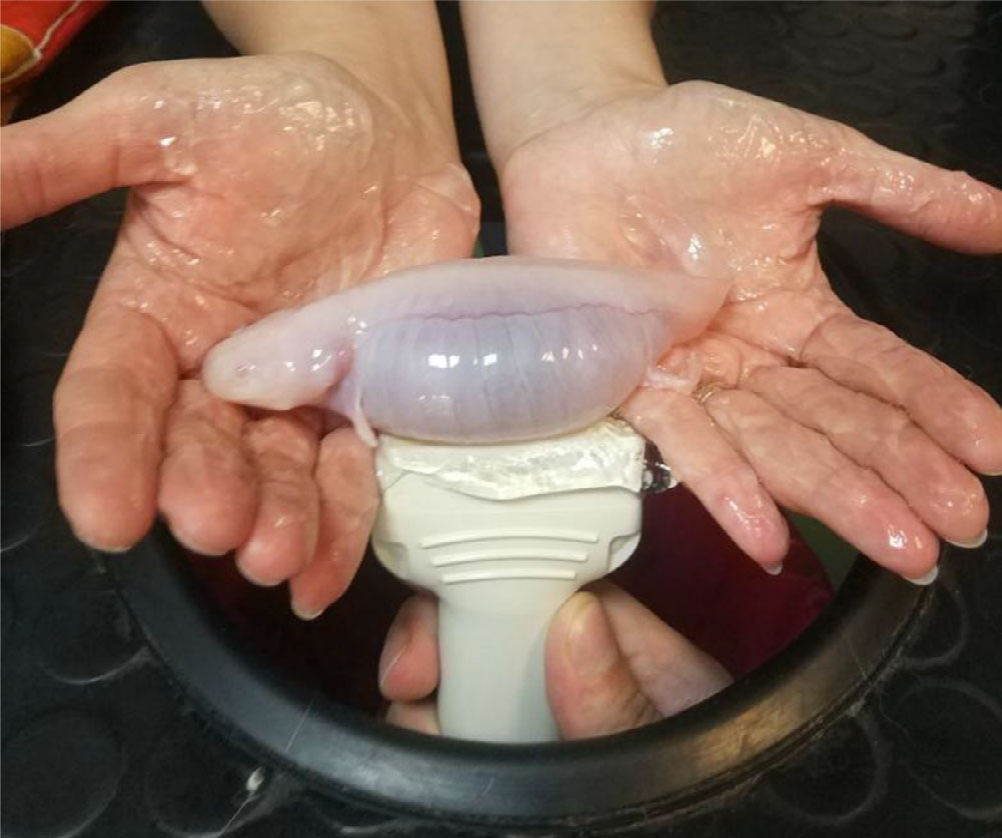
The staff hand is moistened with ultrasound gel to avoid damaging the skin integrity before ultrasonographic imaging of the patient axolotl.

**FIGURE 5:**
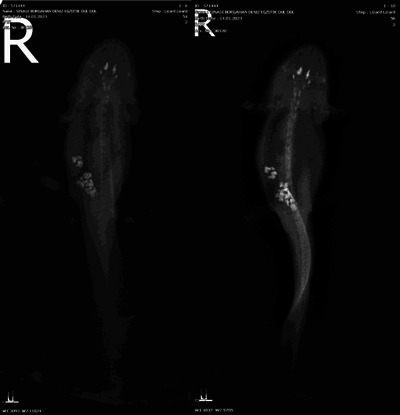
Volume increase in the coelomic cavity of the patient axolotl by radiographic examination at different contrasts.

**FIGURE 6:**
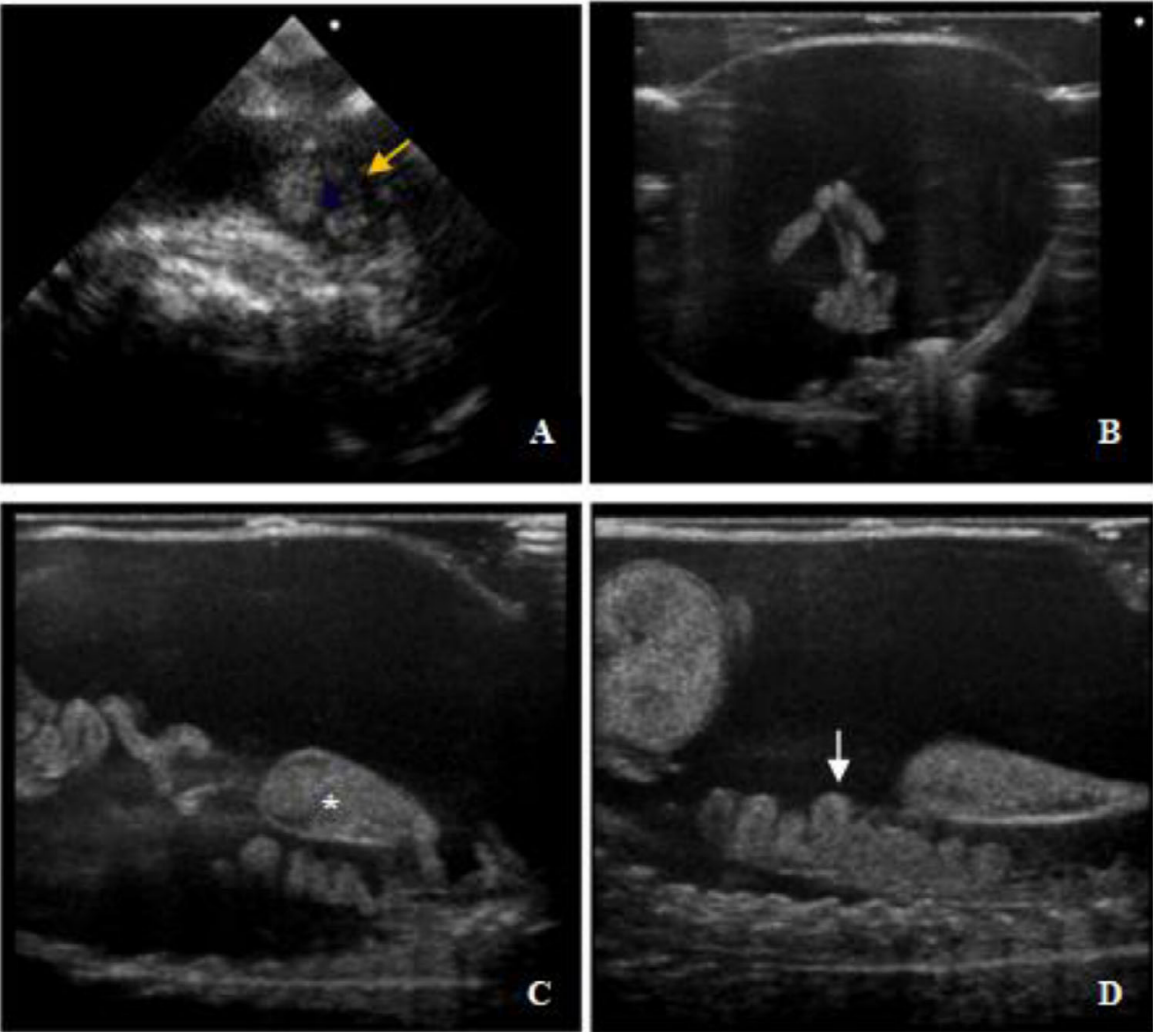
Ultrasonographic images of the coelomic cavity of the patient axolotl with hydrocoelom. (A) Longitudinal image of the heart (heart is indicated by the yellow arrow), (B) transversal image of the intestine, (C, D) dorsal positioning of the intestine and urinary bladder (urinary bladder is indicated by a white star and intestines by a white arrow).

To prevent skin damage during patient contact, the personnel moistened their hands with ultrasound gel (Figure 4). Again, the personnel performed the examination in short periods to prevent skin drying. During the clinical examination, we observed an increased volume in the coelomic cavity and palpable stretches in the abdominal wall. Radiographic views of the coelomic cavity were monitored under an x‐ray system with double detectors (amorphous silicon with caesium iodide scintillators, 2500 × 3072 pixels). We detected radiolucent heterogeneous areas caudal (Figure 5). Ultrasonographic evaluation was performed for the purpose of definitive diagnosis, and fluid accumulation was confirmed in the coelomic cavity. It was observed that the coelomic organs were pushed to the dorsal side of the body because of the accumulated fluid, and no pathology was detected in echocardiographic imaging (Figure 6).

The coelomic cavity was punctured with a 26G insulin injector through USG guidance, and 10 cc of content was drained. The clear and odourless content obtained by this coelomic paracentesis procedure was sent for microbiological and cytological analysis.

The water temperature was reduced to 15°C–20°C, which is optimal for axolotls and was followed daily. The replacement water was decided to be high pH drinking water to bring the aquarium acidity values to the ideal level for axolotl (pH 7–7.5). The volume of water in the aquarium was regulated to be more than 2 L/axolotl so that the animal could be completely surrounded, and there was no dry area (Duhon 2018). A 12/12‐h light/dark cycle was established to minimize stress.

The diet was varied, including earthworms, frozen brine shrimp, trout pellets and beef liver or heart. Foods were given three times a week in 3–5 mm sizes until satiated, with a maximum of five pellets at a time (Canadian Council on Animal Care 2011b; Khattak et al. 2014).

Similar to amphibians, axolotls can receive drug administration subcutaneously, intra‐coelomically, orally or topically through skin impregnation (Gentz 2007). Antibiotic (enrofloxacin 5–10 mg/kg, PO, q24 h, Baytril 5%, Bayer, Turkey) was administered orally to prevent infections. Since the efficacy of diuretic use in the treatment of hydrocoelom has been unclear in previous studies (Clancy et al. 2015), diuretic use was not included in the treatment in this case. Microbiologic examination of ascites fluid was evaluated as non‐infective. Antibiotic treatment was after 1 week, and the arrangements in the habitat were maintained.

**FIGURE 7:**
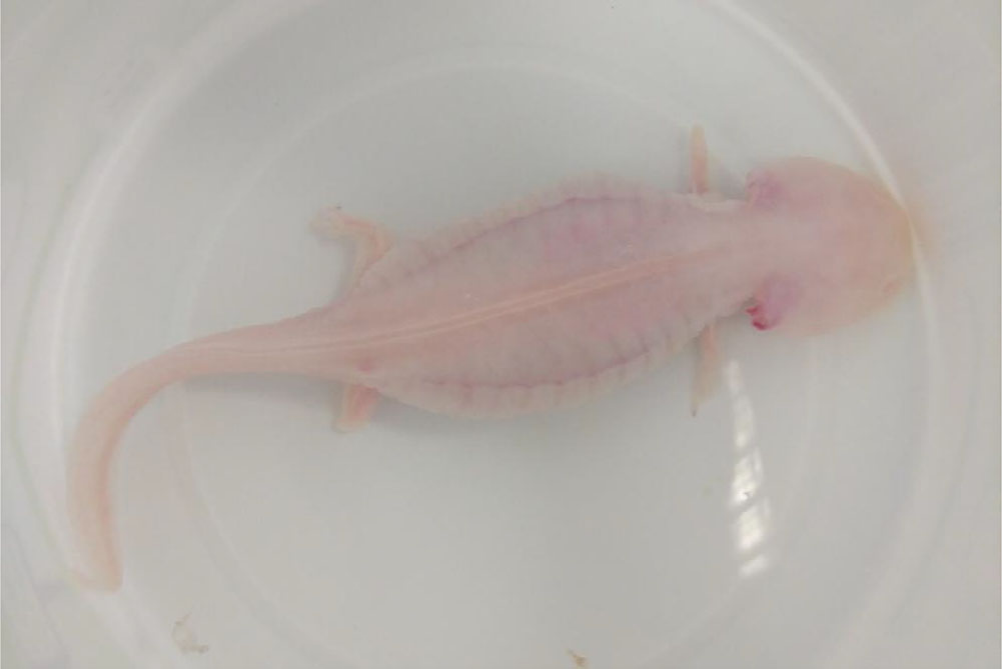
After treatment of hydrocoelom, the axolotl's abdominal swelling returned to normal and the short right forelimbs and hind limbs were remodelled and the external gills were lengthened.

The water in the aquarium was maintained at 18°C–20°C and pH 7. Re‐examinations showed an improved prognosis occurred in Weeks 1 and 3 after discharge. There was remodelling of the previously lost right forelimbs and hind limbs and elongation of the external gills (Figure 7). The last follow‐up examination occurred 6 months after treatment. The owner reported normal behaviour, a good appetite and a significant enhancement in activity.

## 3 Discussion and Conclusion

Hydrocoelom is defined as an enlargement in the coelomic cavity with fluid. This disorder is common in amphibians; in fact, it was the most common disorder in axolotls in a retrospective case study, followed by buoyancy diseases and bacterial dermatitis (Takami and Une 2017). Case success in these patients is generally determined as 36.5% (Clancy et al. 2015). Its aetiology includes infectious causes, injuries and various health problems. Faults in axolotl breeding are also significant factors that cause such diseases in amphibian species (Ortiz‐Ordoñez et al. 2016; Roe et al. 2006; Uygur et al. 2023). This case report describes the management of hydrocoelom in an axolotl with abdominal enlargement and weakness. The above‐discussed points have been taken into consideration during the examinations.

In hydrocoelom examination, infections must definitely be considered. Fluid aspiration for culture is considered the most important antemortem diagnostic method to differentiate infectious causes (Clancy et al. 2015). Reports indicate that treating hydrocoelom with enrofloxacin increases case success more than five times (Borland 2000; Clancy et al. 2015; Gentz 2007). Enrofloxacin is known to have a favourable effect on survival, whereas the efficacy of diuretics in the treatment of hydrocoelom is unknown. In the present case, the cause of increased volume in the abdominal cavity of an axolotl was investigated, and infectious causes were excluded from the differential diagnosis based on laboratory results. A 1‐week antibiotic treatment was started for prophylaxis owing to its favourable effect on infections (Borland 2000; Clancy et al. 2015; Gentz 2007).

Organ abnormalities were also recorded as the cause of ascites, such as abnormal heart development in axolotls (Fransen and Lemanski 1989; Lemanski 1973), liver disease in amphibians (Crawshaw and Weinkle 2000) and renal failure in American bullfrogs (Jacobson et al. 2004). As noted, fluids from failed organs, lymph surrounding wounds or regenerating tissues may accumulate in the coelomic cavity. Physical examination, radiographic imaging, ultrasonographic evaluation and echocardiographic imaging evidenced (i) no injury or organ abnormality and (ii) coelomic fluid accumulation.

Increased environmental temperature and acidity in water pH are considered a further stress factor in axolotls (Canadian Council on Animal Care 2011b; Harvey Pough 2007; McMenamin and Hadly 2010; Robb and Toews 1987) and a probable cause of hydrocoelom. Due to the high permeability of their skin, suboptimal aquarium water leads to metabolic and circulatory disorders, damage to organs and consequently diseases (Baitchman 2008; Gentz 2007). Amphibians have thin, permeable skin that allows the exchange of oxygen and carbon dioxide, and the movement of water in and out of the body. For this reason, the quality of the water in which they live is a factor that greatly affects the health of the amphibians. Axolotls should be kept in water with a temperature of 15°C–20°C and a pH range of 7–7.5 (Canadian Council on Animal Care 2011a; Farkas and Monaghan 2015; Huang et al. 2017). Chlorine damages the protective mucous layer covering the skin of amphibians, such as axolotls, and may predispose these animals to infection (Reed 2005). Therefore, the water of an aquarium must not contain chlorine and chloramine (Canadian Council on Animal Care 2011a). Unsuitable aquarium conditions were considered as the primary cause of the disease, and the habitat was ideally rearranged. The water temperature was fixed at 18°C–20°C and pH 7. Until improvement was observed, 20% of the aquarium water was changed daily with unchlorinated drinking water and then the treatment device was switched. At the end of the second week, fluid accumulation did not occur again in the patient's coelomic cavity.

The prognosis was satisfactory with this treatment technique. The treatment protocol did not include diuretics, because recovery was achieved rapidly with the aspiration of the coelomic fluid, antibiotic treatment and environmental arrangement. Since therapy resolved and follow‐up did not indicate a relapse, we concluded that environmental stress was the cause.

Despite the clinical prevalence of hydrocoelom in amphibians, there are few well‐documented case reports for hydrocoelom (Frye 1992; Tarigo et al. 2006; Vannevel 2006; Vaughan et al. 2006). Moreover, they did not investigate the cause of hydrocoelom in axolotls. For instance, it was studied by Tarigo et al. (2006) that an adult female albino South African Clawed frog (*Xenopus laevis*) showed signs of depression, but the study only used mycobacterial culture and cytologic monitoring to make a diagnosis. Takami and Une (2017) reported hydrocoelom in axolotls but did not investigate probable causes. Vieu et al. (2024) presented ultrasonographic images of only healthy axolotls. No paper so far could present an investigation of hydrocoelom in axolotls.

Our study is the first full‐fledged presentation of an examination of an axolotl with hydrocoelom. It considered all known possible causes and eliminated infection and injury swiftly from the list. Rearranging unsuitable aquarium conditions appeared effective at the patient's treatment. It serves as a valuable resource for clinicians managing cases of hydrocoelom in axolotls.

## Author Contributions

**Şeyma Sueda Cirit**: conceptualization, data curation, formal analysis, funding acquisition, investigation, methodology, project administration, resources, supervision, validation, visualization, writing – original draft. **Şinasi Borgahan Deniz**: investigation, methodology, visualization. **Burhan Cirit**: investigation. **Abdulahad Bayraktar**: data curation, funding acquisition, supervision, writing – original draft, writing – review and editing.

## Ethics Statement

An ethical statement was received from the authors that the data, information and documents presented in this article were obtained within the framework of academic and ethical rules and that all information, documents, evaluations and results were presented in accordance with scientific ethics and moral rules.

## Conflicts of Interest

The authors declare no conflicts of interest.

## Data Availability

The data that support the findings of this study are available from the corresponding author upon reasonable request.
